# Epitope specificity determines cross‐protection of a SIT‐induced IgG_4_ antibody

**DOI:** 10.1111/all.12710

**Published:** 2015-09-30

**Authors:** E. Gadermaier, L. K. James, M. H. Shamji, K. Blatt, K. Fauland, P. Zieglmayer, T. Garmatiuk, M. Focke‐Tejkl, M. Villalba, R. Beavil, W. Keller, P. Valent, S. R. Durham, H. J. Gould, S. Flicker, R. Valenta

**Affiliations:** ^1^ Division of ImmunopathologyDepartment of Pathophysiology and Allergy ResearchCentre for Pathophysiology, Infectiology and ImmunologyVienna General HospitalMedical University of ViennaViennaAustria; ^2^ Randall Division of Cell and Molecular BiophysicsKing's College LondonLondonUK; ^3^ Allergy and Clinical ImmunologyNational Heart and Lung InstituteImperial College LondonLondonUK; ^4^ Division of Hematology and HemostaseologyDepartment of Internal Medicine IVienna General HospitalMedical University of ViennaViennaAustria; ^5^ Institute of Molecular BiosciencesUniversity of GrazGrazAustria; ^6^ Vienna Challenge ChamberAllergy Centre Vienna WestViennaAustria; ^7^ Departamento de Bioquımica y Biologıa Molecular IUniversidad Complutense de MadridMadridSpain

**Keywords:** calcium‐binding protein, cross‐reactivity, pollen allergen, recombinant allergen, SIT‐induced IgG antibody

## Abstract

**Background:**

The calcium‐binding 2EF‐hand protein Phl p 7 from timothy grass pollen is a highly cross‐reactive pollen pan‐allergen that can induce severe clinical symptoms in allergic patients. Recently, a human monoclonal Phl p 7‐specific IgG_4_ antibody (mAb102.1F10) was isolated from a patient who had received grass pollen‐specific immunotherapy (SIT).

**Methods:**

We studied epitope specificity, cross‐reactivity, affinity and cross‐protection of mAb102.1F10 towards homologous calcium‐binding pollen allergens. Sequence comparisons and molecular modelling studies were performed with ClustalW and SPADE, respectively. Surface plasmon resonance measurements were made with purified recombinant allergens. Binding and cross‐reactivity of patients' IgE and mAb102.1F10 to calcium‐binding allergens and peptides thereof were studied with quantitative RAST‐based methods, in ELISA, basophil activation and IgE‐facilitated allergen presentation experiments.

**Results:**

Allergens from timothy grass (Phl p 7), alder (Aln g 4), birch (Bet v 4), turnip rape (Bra r 1), lamb's quarter (Che a 3) and olive (Ole e 3, Ole e 8) showed high sequence similarity and cross‐reacted with allergic patients' IgE. mAb102.1F10 bound the C‐terminal portion of Phl p 7 in a calcium‐dependent manner. It cross‐reacted with high affinity with Ole e 3, whereas binding and affinity to the other allergens were low. mAb102.1F10 showed limited cross‐inhibition of patients' IgE binding and basophil activation. Sequence comparison and surface exposure calculations identified three amino acids likely to be responsible for limited cross‐reactivity.

**Conclusions:**

Our results demonstrate that a small number of amino acid differences among cross‐reactive allergens can reduce the affinity of binding by a SIT‐induced IgG and thus limit cross‐protection.

**Abbreviations**
BSA: bovine serum albumin
SAS: solvent‐accessible surface
SIT: specific immunotherapy

Referring to the WHO/IUIS Allergen Nomenclature, allergens are listed according to their origin and thus annotated to the individual allergen sources 1. However, it has turned out to be useful to group allergens into families which are related in terms of their sequence, three‐dimensional structure and immunological cross‐reactivity 2, 3. The latter grouping of allergens is extremely useful to explain clinical syndromes based on the cross‐reactivity of patients' IgE antibodies and/or T‐cell receptors with structurally related allergens from different allergen sources. For example, cross‐reactivity of the major birch pollen allergen Bet v 1 with counterparts in various plant foods is responsible for oral allergy syndrome and exacerbations of atopic eczema in birch pollen‐allergic patients upon ingestion of plant food containing cross‐reactive allergens 4, 5, 6. Other highly cross‐reactive plant allergens are the profilins, which are ubiquitous cytoskeletal proteins 7, 8, 9, 10, and the polcalcins, a family of calcium‐binding allergens which may cause clinical reactions to multiple plant pollens because they occur in pollens of most plants and share a high degree of sequence similarity 11, 12, 13, 14. Polcalcins from grass pollen seem to be the most potent primary sensitizers, and sensitized patients show extensive cross‐reactivity of their IgE antibodies with related allergens in pollens of various grasses, trees and weeds 15, 16. Although there is extensive cross‐reactivity of allergic patients' IgE antibodies towards allergens from the Bet v 1, profilin and polcalcin family, it is a matter of debate whether immunotherapy with one member of a particular allergen family cross‐protects against members of other allergen sources. For example in a double‐blind study of subcutaneous immunotherapy that employed a ragweed extract in grass–ragweed dual‐sensitized individuals, the extract proved highly effective during the ragweed season but with no cross‐protection being observed during the grass allergy season 17. A similar question is whether birch pollen extract‐based SIT has a protective effect on oral allergy syndrome (OAS) to apple or hazelnut 18, 19, 20, 21. Some trials reported no 19 or no significant 18 effects of birch pollen SIT to apple allergy in comparison with placebo, while other authors reported positive effects at varying degrees 20, 21. A more recent study indicated that during birch pollen SIT in most, but not all, food‐sensitized patients, there was an induction of allergen‐specific IgG_4_ responses 22. These findings are supported by another trial showing that food‐tolerant individuals had significantly higher Mal d 1 (apple)‐ and Cor a 1 (hazelnut)‐specific IgG_4_/IgE ratios in comparison with individuals with food allergy 23.

It is thus possible that the levels and qualities (*i.e*. cross‐reactivity, affinities) of therapy‐induced IgG antibodies may be important for cross‐protection during SIT.

In this study, we analysed in detail a human monoclonal IgG_4_ antibody, mAb102.1F10, specific for polcalcin from timothy grass pollen, Phl p 7, which was isolated by molecular cloning from a single isolated B cell from a grass pollen‐allergic patient who had received grass pollen SIT 24. mAb102.1F10 was shown to bind with high affinity to Phl p 7 and to inhibit allergic patients' IgE binding to Phl p 7 as well as Phl p 7‐induced basophil activation and IgE‐facilitated allergen presentation 24. Here, we investigated the cross‐reactivity of mAb102.1F10 with related calcium‐binding allergens from tree and weed pollens, determined its affinity towards the cross‐reactive allergens and mapped its binding site on Phl p 7. Our results reveal a very limited cross‐reactivity of mAb102.1F10. Only few exchanges of surface‐exposed amino acids on the mAb102.1F10 binding site expressed by cross‐reactive allergens appear necessary to prevent its avid binding to alternative plant sources of polcalcins related to Phl p 7. Thus, epitope specificities that lead to different affinities of binding of SIT‐induced IgG may be critical for cross‐protection.

## Materials and methods

### Recombinant allergens, synthetic peptides, antibodies, antisera and patients sera

Recombinant timothy grass pollen allergen Phl p 7 15 was obtained from Biomay AG (Vienna, Austria). Recombinant Ole e 8 25 from olive pollen and Bra r 1 from turnip rape pollen were expressed in *Escherichia coli* and purified by nickel‐affinity chromatography 26. Recombinant Che a 3 27 from lamb's‐quarters' pollen was expressed and purified as described 28. Recombinant Bet v 4 29 from birch pollen, Aln g 4 30 from alder pollen and Ole e 3 31 from olive pollen were cloned into the bacterial expression vector pET151 (Life Technologies, Carlsbad, CA, USA) and expressed in BL21 star (DE3) cells. The protein was purified using HisTrap FF crude columns (GE Healthcare, Little Chalfont, UK), followed by size exclusion chromatography using an S200 column (GE Healthcare). Two synthetic peptides that span the *N*‐terminal EF‐hand 1 (peptide 1) and *C*‐terminal EF‐hand 2 (peptide 2) of Phl p 7 (aa 2–37, aa 37–78) were synthesized 32.

Recombinant Phl p 7‐specific IgG_4_ (mAb102.1F10) was isolated from a SIT‐treated grass pollen‐allergic patient and purified as described 24, 33. Rabbit anti‐Phl p 7, anti‐peptide 1 and anti‐peptide 2 antisera were obtained by immunization of rabbits 32.

Sera from grass pollen‐allergic patients with sensitization to Phl p 7 were analysed in an anonymized manner with permission from the Ethics Committee of the Medical University of Vienna (EK641/2014).

### Quantitative binding of allergic patients' IgE and mAb102.1F10 to EF‐hand allergens

**Figure 1:**
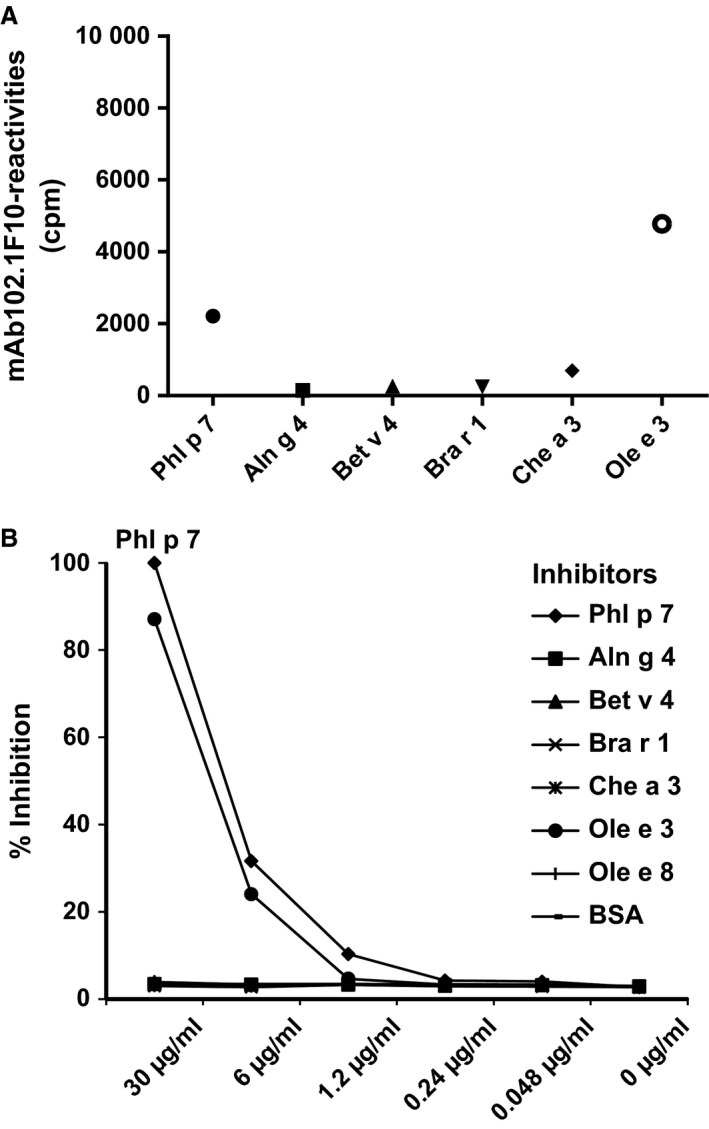
Cross‐reactivity of mAb102.1F10 with Phl p 7 and related EF‐hand allergens. (A) mAb102.1F10 reactivities to nitrocellulose‐dotted 2EF‐hand allergens (*x*‐axis: Phl p 7, Aln g 4, Bet v 4, Bra r 1, Che a 3, Ole e 3) are shown as absolute cpm values (*y*‐axis). (B) Specific inhibition of mAb102.1F10 binding (*y*‐axis: % inhibition) to Phl p 7 after pre‐incubation with inhibitors (*x*‐axis: Phl p 7, Aln g 4, Bet v 4, Bra r 1, Che a 3, Ole e 3, Ole e 8, BSA; 30, 6, 1.2, 0.24, 0.048, 0 μg/ml).

EF‐hand allergens (Phl p 7, Aln g 4, Bet v 4, Bra r 1, Che a 3, Ole e 3, Ole e 8) and BSA (negative control) were dotted to nitrocellulose strips (1 μg/dot) (Schleicher & Schuell, Dassel, Germany) and incubated with sera from 14 Phl p 7‐allergic patients (diluted 1 : 5), with serum from a nonallergic individual, with mAb102.1F10 and control IgG_4_ (1 μg/strip**;** Sigma‐Aldrich, St. Louis, MO, USA) and with Phl p 7‐specific rabbit antiserum and control antiserum raised against Der p 2 from house dust mite. Bound human IgG_4_ antibodies were detected with a mouse monoclonal anti‐human IgG_4_ antibody (PharMingen, San Diego, CA, USA), followed by a ^125^I‐labelled rabbit anti‐mouse antiserum (Perkin Elmer, Waltham, MA, USA), whereas bound rabbit antibodies were detected with ^125^I‐labelled goat anti‐rabbit antisera (Perkin Elmer). Bound patients' IgE antibodies were detected with ^125^I‐labelled anti‐human IgE (BSM Diagnostica, Vienna, Austria) (data not shown). Bound ^125^I‐labelled antibodies were quantified with a gamma counter (Wizzard, Automatic Gamma Counter; Wallac, Uppsala, Sweden), and results represent mean counts per minute (cpm)/dot (Fig. 1A).

### Cross‐reactivity of mAb102.1F10 to Phl p 7 and related EF‐hand allergens

ELISA plates (Nunc; Maxisorp, Roskilde, Denmark) were coated with Phl p 7 (0.1 μg/well in 0.1 m bicarbonate buffer, pH 9.6). After washing with buffer A (PBS/0.05% vol/vol Tween‐20) and blocking with buffer B (PBS/1% wt/vol BSA) 100 μl/well of mAb102.1F10 (0.1 μg/ml in buffer B), which had been pre‐incubated overnight with increasing concentrations (0.048–30 μg/ml in fivefold dilutions diluted in buffer B) of inhibitors (Phl p 7, Aln g 4, Bet v 4, Bra r 1, Che a 3, Ole e 3, Ole e 8, BSA as negative control) was added. Binding of mAb102.1F10 was detected with a horseradish peroxidase (HRP)‐labelled rabbit anti‐human IgG antiserum diluted in buffer B (Dako, Carpenteria, CA, USA). The colour reaction was started by the addition of 1.7 mm 2, 2′azinobis‐[3‐ethyl‐benzothiazoline‐6‐sulfonic acid] (Sigma‐Aldrich) in 60 mm citric acid, 77 mm Na_2_HPO_4_.2H_2_O, 3 mm H_2_O_2_. Plates were incubated in the dark and extinctions (optical density 405 nm) were determined with an ELISA reader. All determinations were performed in duplicates and mean values were calculated. Relative inhibition was calculated in percentage of the maximal inhibition of mAb102.1F10 binding after pre‐incubation with the maximal concentration (30 μg/ml) of Phl p 7 (Fig. 1B).

### IgE‐facilitated allergen binding assay

**Figure 2:**
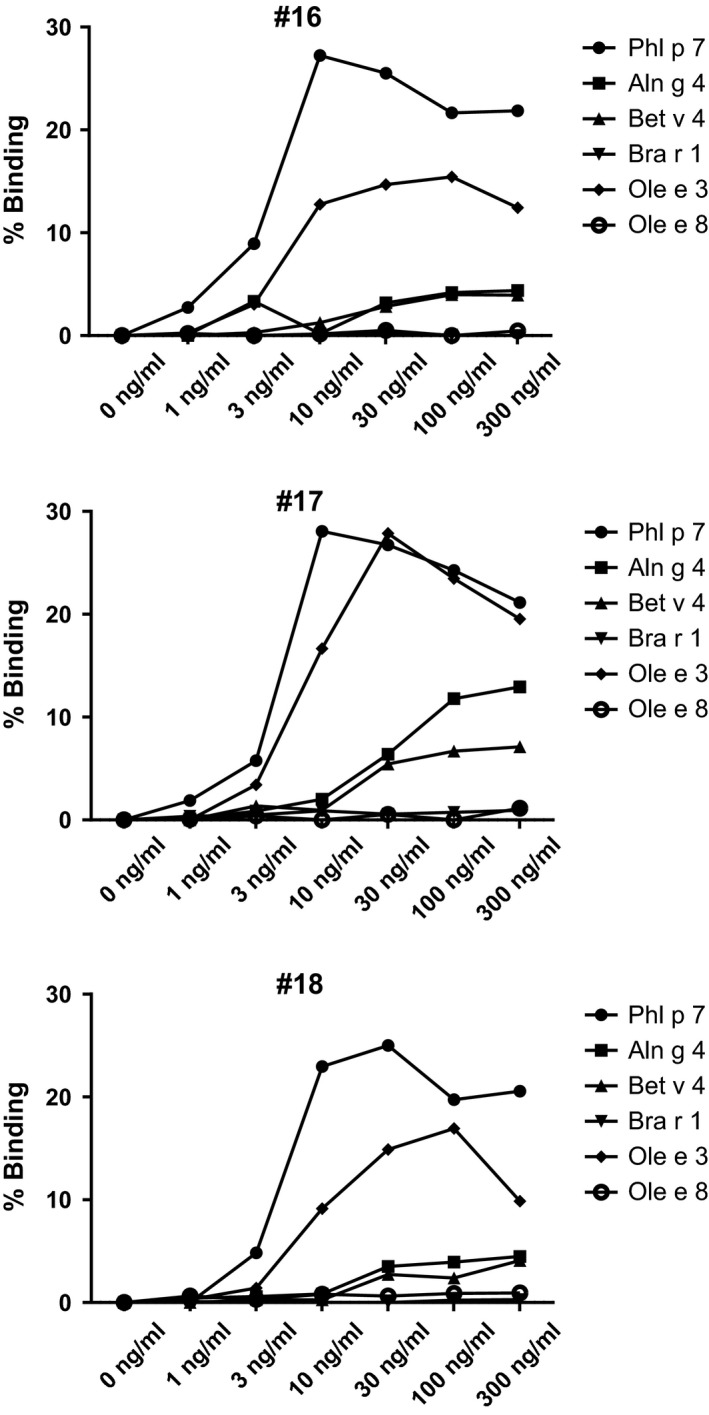
Binding of immune complexes consisting of patients' IgE and EF‐hand allergens to B cells. Relative binding (*y*‐axes: % binding) of complexes consisting of increasing doses (*x*‐axes: 0, 1, 3, 10, 30, 100, 300 ng/ml) of EF‐hand allergens (Phl p 7, Aln g 4, Bet v 4, Bra r 1, Ole e 3, Ole e 8) and patients' serum IgE (patients #16, #17, #18) to CD23‐expressing B cells.

IgE‐facilitated allergen binding to CD23‐expressing B cells was determined as described 34. In brief, EF‐hand allergens (Phl p 7, Aln g 4, Bet v 4, Bra r 1, Ole e 3, Ole e 8; 0–300 ng/ml) were incubated in triplicates with sera from Phl p 7‐allergic patients (patients #16, 17, 18; Fig. 2) to allow IgE–allergen complex formation. EBV‐transformed B cells (100.000 cells/sample) were added and the mixture was incubated at room temperature. IgE binding to the cells was detected with phycoerythrin (PE)‐labelled anti‐human IgE (MACS; Miltenyi Biotec, Bergisch Gladbach, Germany) by flow cytometry, and relative binding of mean values is displayed.

### Inhibition of allergic patients' IgE reactivity to Phl p 7 and related EF‐hand allergens with mAb102.1F10

**Figure 3:**
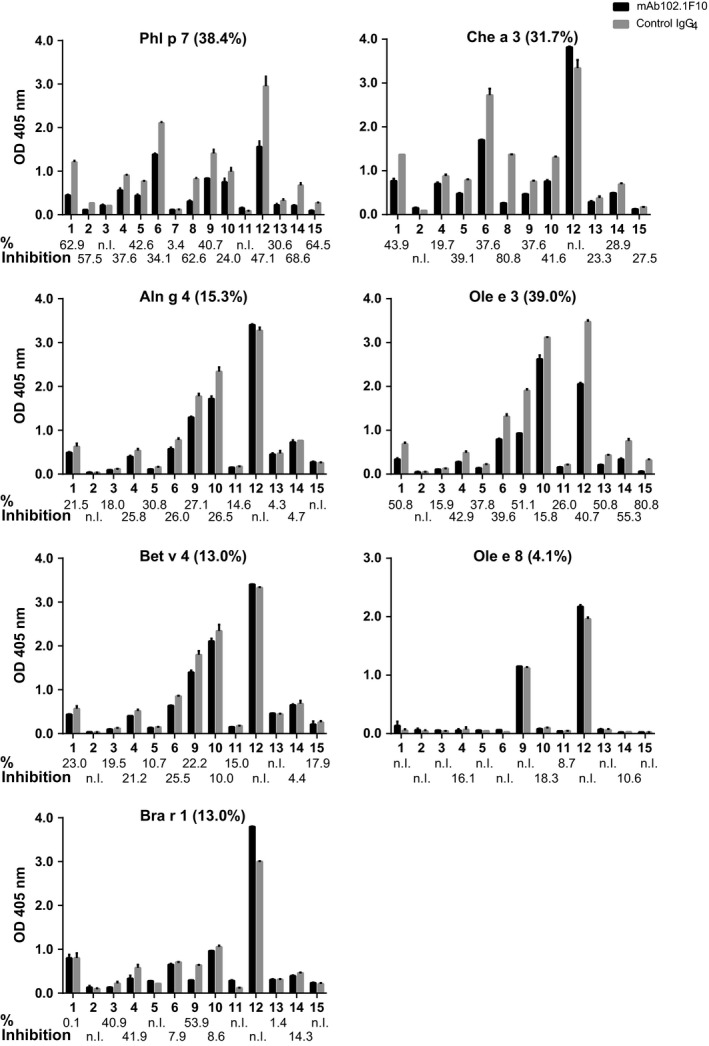
Inhibition of Phl p 7‐allergic patients' IgE binding to Phl p 7 and related EF‐hand allergens by mAb102.1F10. Patients' IgE bindings (*y*‐axes: OD values at 405 nm ± SD) to plate‐bound Phl p 7, Aln g 4, Bet v 4, Bra r 1, Che a 3, Ole e 3 and Ole e 8 after pre‐incubation with mAb102.1F10 (black bars) or control IgG_4_ (grey bars) are displayed for 15 Phl p 7‐allergic patients (ID 1–15) (*x*‐axes). Percentage inhibitions are shown on the bottom of each chart (n.I.: no Inhibition). Mean percentage inhibitions for each allergen are indicated in parentheses in the headline of each chart.

ELISA plates were coated with allergens (0.1 μg/well) and pre‐incubated with mAb102.1F10 (2 μg/well) or with control IgG_4_ (2 μg/well; Sigma‐Aldrich). Next, plates were incubated with sera from Phl p 7‐allergic patients or a nonallergic subject (diluted 1 : 5). Bound IgE antibodies were detected with a mouse monoclonal anti‐human IgE antiserum (PharMingen) followed by the HRP‐labelled sheep anti‐mouse antiserum (GE Healthcare). All determinations were performed in duplicates and mean values were calculated. Percentage inhibitions were determined as follows: 100‐(OD bound IgE after pre‐incubation with mAb102.1F10 * 100/OD bound IgE after pre‐incubation with control IgG_4_) (Fig. 3).

### Indirect epitope mapping of mAb102.1F10

**Figure 4:**
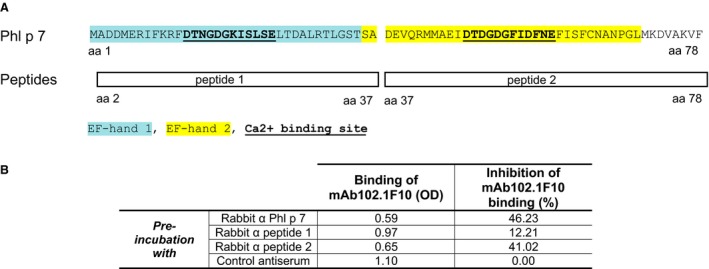
Epitope mapping of mAb102.1F10. (A) Amino acid (aa) sequences of Phl p 7 (aa 1–78) and of Phl p 7 peptides (peptide 1: aa 2–37; peptide 2: aa 37–78) are shown. EF‐hand domains are coloured (EF‐hand 1: blue; EF‐hand 2: yellow) and calcium binding sites are bold and underscored. (B) Inhibition of mAb102.1F10 binding to Phl p 7 obtained by pre‐incubation of Phl p 7 with rabbit antisera raised against Phl p 7, peptide 1, peptide 2, or with a control rabbit antiserum. Reaction levels of mAb102.1F10 binding to Phl p 7 after pre‐incubation with rabbit antisera (OD levels at 405 nm) and percentage inhibition of mAb102.1F10 binding in relation to pre‐incubation with the control antiserum are shown.

ELISA plate‐bound Phl p 7 (0.1 μg/well) was pre‐incubated with rabbit antisera (1 : 20 in buffer B) raised against Phl p 7, Phl p 7 peptides 1 or 2 (Fig. 4A) (inhibitors), or a normal rabbit serum (control). After washing, mAb102.1F10 or control IgG_4_ (Sigma‐Aldrich) (0.1 μg/well) was added and bound human IgG antibodies were detected with HRP‐labelled rabbit anti‐human IgG antibodies (Dako). All determinations were performed in duplicates, mean ODs were calculated, and percentage inhibitions were calculated according to the formula: 100 – (OD mAb102.1F10 binding after pre‐incubation with inhibitors * 100/OD mAb102.1F10 binding after pre‐incubation with control) (Fig. 4B).

### Sequence alignment of Phl p 7 with related EF‐hand allergens

**Figure 5:**
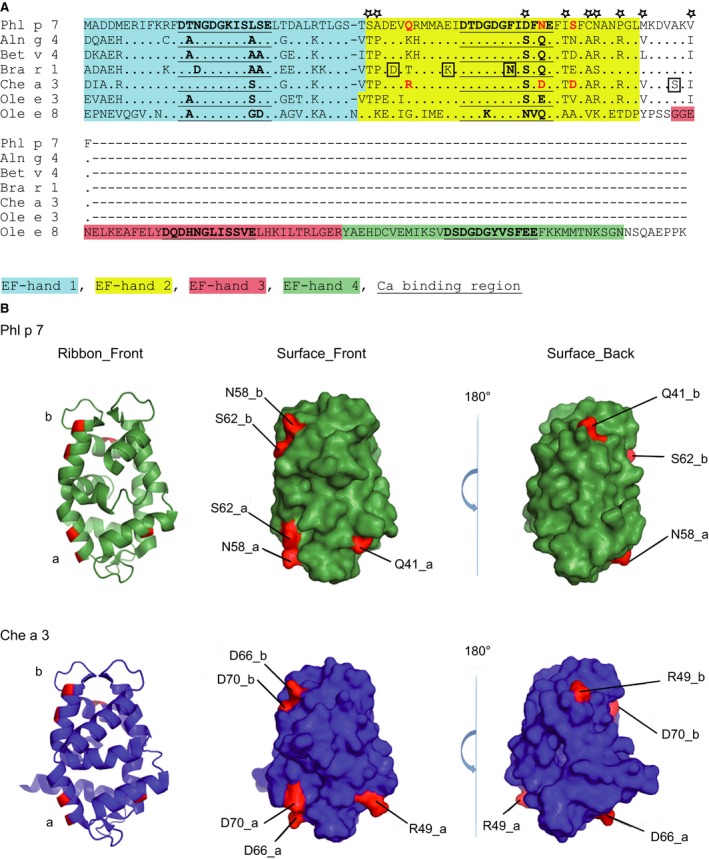
(A), Protein sequence alignment of Phl p 7 with related EF‐hand allergens. Identical amino acids are indicated by dots and gaps are indicated by dashes. EF‐hand domains are coloured (EF‐hand 1: blue; EF‐hand 2: yellow; EF‐hand 3: red; EF‐hand 4: green) and calcium binding sites are bold and underscored. Amino acids that according to epitope mapping may influence binding of mAb102.1F10 are highlighted in red. Amino acids that were excluded to contribute to differences in binding are boxed or indicated by asterisks. (B) Ribbon and surface representation of Phl p 7 and Che a 3 dimers (chain a and chain b). Residues possibly involved in binding of mAb102.1F10 (see Fig. 5A, red) are highlighted on the molecular surface of Phl p 7 (green) and of Che a 3 (blue). The dimer structures are shown as ribbon and as surface representation in the same orientation (Surface_Front) and rotated 180° about the *y*‐axes (Surface_Back).

Sequences of Phl p 7 (Swiss‐Prot: O82040.1), Aln g 4 (Swiss‐Prot: O81701.1), Bet v 4 (GenBank: CAA73147.1), Bra r 1 (Swiss‐Prot: P69197.1), Che a 3 (Swiss‐Prot: Q84V36.1), Ole e 3 (Swiss‐Prot: O81092.1) and Ole e 8 (Swiss‐Prot: Q9M7R0.1) were aligned with clustalw2 35 and revised by hand editing (Fig. 5A). Amino acid sequence identities were calculated (Table S1) 35, and EF‐hand domains and Ca^2+^ binding sites were indicated according to UniProt entries.

### Surface exposure analysis of Che a 3 and Phl p 7

The relative surface accessibility of individual amino acids was calculated for both monomers of the dimer structures using SPADE 36. For Phl p 7, the coordinates PDB:1K9U were used, and for Che a 3, the coordinates PDB:2OPO (chains a and b) were used. The absolute surface exposure (in Å^2^) as well as the relative surface exposure (in % of the maximal surface exposure of the individual residue) was calculated, along with the average values from chains a and b (Table 1).

**Table 1:** Solvent‐accessible surface (SAS) of variable residues (see Fig. 5A, red) is shown for Phl p 7 (top) and Che a 3 (bottom) for both monomer and dimer structure with the absolute SAS (in Å^2^) as well as the relative surface exposure in the dimer (in % of the maximal surface exposure of the individual residue), along with the average values calculated from both chains a and b

Chain a			Chain b			Average	
AA	SAS [A^2^]	SAS [%]	AA	SAS [A^2^]	SAS [%]	SAS [A^2^]	SAS [%]
Phl p 7							
Q41_a	62.896	28.6	Q41_b	96.964	44.1	79.930	36.4
N58_a	103.388	55.3	N58_b	114.858	61.5	109.123	58.4
S62_a	60.665	40.1	S62_b	62.490	41.3	61.578	40.7
Che a 3							
R49_a	181.045	62.8	R49_b	139.131	48.3	160.088	55.6
D66_a	102.104	57.7	D66_b	101.834	57.5	101.969	57.6
D70_a	85.088	48.0	D70_b	84.678	47.8	84.883	47.9

## Results

### mAb102.1F10 shows different affinity to cross‐reactive EF‐hand allergens

Table S1 shows that the sequence identity among six 2EF‐hand allergens (Phl p 7, Aln g 4, Bet v 4, Bra r 1, Che a 3, Ole e 3) was high and ranged between 67 and 91% (mean: 75.8%). Only the 4EF‐hand allergen Ole e 8 showed lower sequence identity (38–48%, mean: 43%) due to its different length and architecture (Table S1).

The affinities of mAb102.1F10 to the purified EF‐hand allergens were measured by surface plasmon resonance measurements. Phl p 7 was recognized with highest affinity (K_D_: 2.11 × 10^−9^ m) closely followed by Ole e 3 (K_D_: 6.18 × 10^−9^ m) which shares 68% sequence identity with Phl p 7 (Table S2). Interestingly, Aln g 4, Bet v 4 and Bra r 1, which share a similar degree of sequence identity to Phl p 7 (*i.e*. 67–69%) as Ole e 3, were recognized with approximately 1000 times lower affinity (K_D_s: Aln g 4: 7.93 × 10^−6^ m; Bet v 4: 6.26 × 10^−6^ m; Bra r 1: 6.57 × 10^−6^ m) (Table S2). Ole e 8 was bound with lowest affinity (K_D_~7.06 × 10^−5^ m) (Table S2).

### Binding of mAb102.1F10 to the EF‐hand allergens depends on the presence of protein‐bound calcium

Allergic patients' IgE reactivity to EF‐hand allergens strongly depends on protein‐bound calcium 11. We therefore investigated whether the binding of mAb102.1F10 to EF‐hand allergens is also calcium sensitive. The binding of mAb102.1F10 to nitrocellulose‐dotted EF‐hand allergens in the presence of calcium reflected the surface plasmon resonance experiments, showing that Phl p 7 and Ole e 3 reacted most strongly whereas the binding to Aln g 4, Bet v 4, Bra r 1 and Che a 3 was more than 100‐fold less (Table S3). Ole e 8 showed no reactivity over the negative controls (*i.e*. BSA, control IgG_4_) (Table S3). Depletion of calcium by addition of EGTA reduced binding of mAb102.1F10 between 33.2% (Ole e 3) and 91.3% (Phl p 7) (Table S3).

### mAb102.1F10 shows limited cross‐reactivity with EF‐hand allergens, whereas allergic patients' IgE antibodies show broad cross‐reactivity

In the dot blot assay, mAb102.1F10 strongly cross‐reacted with Phl p 7 and Ole e 3 and only weakly with Che a 3 > Bet v 4 > Bra r 1 > Aln g 4 (Fig. 1A). Next, we pre‐incubated mAb102.1F10 with increasing doses of EF‐hand allergens and tested for remaining reactivity to Phl p 7 in a competitive inhibition ELISA (Fig. 1B). Only pre‐incubation with Phl p 7 and Ole e 3 completely blocked the binding of mAb102.1F10 to Phl p 7, whereas pre‐incubation with the other EF‐hand allergens hardly influenced its binding to Phl p 7 (Fig. 1B). By contrast, Phl p 7‐allergic patients' polyclonal IgE antibodies showed extensive cross‐reactivity with 2EF‐hand allergens (mean IgE levels: Ole e 3 > Bet v 4 > Aln g 4 > Che a 3 and to a lower extent to Bra r 1) (data not shown). The functional relevance of the cross‐reactivity of allergic patients' IgE with the EF‐hand allergens was demonstrated using the IgE‐facilitated allergen binding assay where the formation of allergen–IgE complexes and their subsequent binding to CD23 on B cells were studied. IgE immune complexes formed with Phl p 7 and Ole e 3 gave the strongest B‐cell staining (maximum 25% binding, Fig. 2). There was also staining with Aln g 4 and Bet v 4 but only at high allergen concentrations and to a lesser extent (maximum 5–15% binding, Fig. 2).

### Cross‐inhibition of patients' IgE binding to EF‐hand allergens by mAb102.1F10 reflects its affinity and cross‐reactivity

When we pre‐incubated EF‐hand allergens with mAb102.1F10**,** variable degrees of inhibition of allergic patients' polyclonal IgE binding were observed (Fig. 3). The inhibition of IgE binding to Phl p 7 (0–68.6%; mean inhibition 38.4%), Ole e 3 (0–80.8%; mean inhibition 39%) and Che a 3 (0–80.8%; mean inhibition 31.7%) by mAb102.1F10 was highest, followed by Aln g 4 (0–30.8%; mean inhibition 15.3%), Bra r 1 (0–53.9%; mean inhibition: 13%) and Bet v 4 (0–25.5%; mean inhibition 13%). Almost no inhibition of IgE binding to Ole e 8 was found.

We also investigated whether mAb102.1F10 can inhibit EF‐hand allergen‐induced basophil activation using blood samples from three Phl p 7‐allergic patients (Fig. S1A–C). mAb102.1F10 inhibited Phl p 7‐ and Ole e 3‐induced basophil activation by up to five times in 2 of 3 patients tested (Fig. S1A, C) but had no effect on basophil activation induced by other EF‐hand allergens (Fig. S1 A–C).

### mAb102.1F10 recognizes the C‐terminal EF‐hand of Phl p 7

To define the binding region of mAb102.1F10 on Phl p 7, we performed inhibition assays using rabbit antisera raised against complete rPhl p 7, the *N*‐terminal peptide 1 or the *C*‐terminal peptide 2 (Fig. 4A) to inhibit the binding of mAb102.1F10 to Phl p 7. We obtained the highest level of inhibition of binding of mAb102.1F10 to Phl p 7 with rabbit antibodies raised against complete Phl p 7 (*i.e*. 46.23%) (Fig. 4B). An almost equal level of inhibition was achieved with rabbit antibodies raised against the C‐terminal portion of Phl p 7 (*i.e*. peptide 2) (Fig. 4B). No relevant inhibition (12.21%) was obtained with a rabbit antiserum specific for the *N*‐terminal peptide 1 or with a control antiserum (Fig. 4B).

### Structural analysis indicates that binding of mAb102.1F10 to EF‐hand allergens depends on a small number of amino acids

Our binding experiments revealed that mAb102.1F10 shows a rather selective recognition of EF‐hand allergens although the degree of overall sequence identity among the EF‐hand allergens was high (*i.e*. approximately 70%) (Table S1). In contrast, allergic patients' IgE reactivity showed broad cross‐reactivity with the EF‐hand allergens (data not shown). We therefore assumed that specificities of recognition may be responsible for the selective binding features of mAb102.1F10. The second EF‐hand domain appeared to be crucial for the binding of mAb102.1F10 (Fig. 4B). We found several amino acid differences between Phl p 7 and EF‐hand allergens that do not contribute to differences in binding because they were identical between a mAb102.1F10‐reactive allergen (*i.e*. Ole e 3) and allergens which bind with lower reactivity to mAb102.1F10 (*i.e*. Aln g 4, Bet v 4, Bra r 1, Che a 3) (Fig. 5A: asterisks, yellow part of sequences). Four amino acid exchanges were observed only in certain allergens (*i.e*. Bra r 1: D, K, N; Che a 3: S; Fig. 5A: boxed) which did not react with mAb102.1F10, but these changes are unlikely to account for the differences in binding because they were not exchanged in the other allergens (*i.e*. Aln g 4, Bet v 4) which did not react with mAb102.1F10. Finally, only three amino acid exchanges remained as possible candidates for the differences in binding (Fig. 5A, B: Phl p 7: Q41, N58, S62 printed in red) because they showed relevant differences regarding their biochemical properties between the allergens with (Phl p 7, Ole e 3) and without (Aln g 4, Bet v 4, Bra r 1, Che a 3) reactivity to mAb102.1F10. We therefore calculated solvent‐accessible surface (SAS) values for each of the three amino acids in the dimeric three‐dimensional structures of Phl p 7 and Che a 3 28, 37 (Fig. 5B) and calculated the average of the corresponding residues, because the conformation of the molecules is slightly different in the two independent monomers of each dimer. By far the largest changes in absolute as well as relative SAS values were observed for the change from Q41 (Phl p 7) to R49 (Che a 3), which predisposed this residue change as the decisive factor for the observed differences (Table 1). The other two residue changes considered could still have an influence due to the change in polarity/net charge, but the change in the surface‐exposed area was much less pronounced.

## Discussion

We have analysed epitope specificity and cross‐reactivity of a monoclonal IgG_4_ antibody, mAb102.1F10, which was isolated from a grass pollen‐allergic patient who had received grass pollen‐specific immunotherapy. mAb102.1F10 was directed against Phl p 7, which belongs to a family of highly cross‐reactive calcium‐binding EF‐hand allergens occurring in pollen of most plants. EF‐hand motifs are sequences in these allergens which contain acidic amino acids needed for the binding of calcium. As described for allergic patients' IgE antibodies, binding of mAb102.1F10 depended on the presence of protein‐bound calcium. In the calcium‐bound form, EF‐hand allergens are supposed to expose amino acids critical for binding of antibodies on the surface of the molecule 26, 30. The binding site of mAb102.1F10 was mapped at the C‐terminal portion of the molecule containing the second EF‐hand.

Allergic patients' IgE showed extensive cross‐reactivity with the Phl p 7‐homologous allergens. By contrast, mAb102.1F10 showed a rather selective binding to Phl p 7 and to Ole e 3, whereas only weak binding to the other EF‐hand allergens was observed with a more than 1000 times lower affinity. Accordingly, mAb102.1F10 mainly inhibited allergic patients' IgE binding and allergen‐induced basophil activation to Phl p 7 and Ole e 3 but had little effect to the other EF‐hand allergens. When we compared the amino acid sequences of the EF‐hand allergens in the binding region of mAb102.1F10, we could, based on the presence or absence of reactivity to the individual EF‐hand allergens, identify three potentially crucial amino acids as candidates to justify differences between binding and nonbinding molecules. According to calculations of the surface exposure of the three identified amino acids in the three‐dimensional structures of Phl p 7 and Che a 3, the latter of which showed highly reduced binding to mAb102.1F10, it could be confirmed that these three amino acids showed different degrees of exposure on the two molecules, rendering them likely candidates for the observed binding differences. Our results thus demonstrate that a monoclonal SIT‐induced IgG antibody showed less cross‐reactivity than allergic patients' IgE antibodies. There are studies indicating that also SIT‐induced polyclonal IgG antibodies show limited cross‐reactivity and thus may be less cross‐protective 22, 38. However, based on our data obtained for a single SIT‐induced IgG and the aforementioned SIT studies showing limited cross‐reactivity of SIT‐induced polyclonal IgG, one still cannot draw general conclusions that SIT‐induced IgG is less cross‐reactive than allergen‐specific IgE. SIT‐induced allergen‐specific IgG antibodies are supposed to contribute to the clinical success of SIT by several mechanisms through inhibition of the IgE–allergen interactions 39. In fact, SIT‐induced allergen‐specific IgG inhibits allergen‐induced basophil and mast cell activation, IgE‐facilitated allergen presentation and boosting of the IgE response. It may therefore be assumed that the limited cross‐reactivity of therapy‐induced IgG will in turn limit cross‐protection against homologous allergens from other sources. However, also other factors besides titre and cross‐reactivity such as avidity and isotype/subclass may play a role in limited cross‐protection.

Our analysis represents only a snapshot of an individual SIT‐induced monoclonal antibody, and the success of SIT will depend on the polyclonal IgG response and thus on the sum and quality of the induced blocking antibodies. However, it provides a useful example and indicates that it is important to determine the sum of epitope specificities, cross‐reactivity, titres, avidities and IgE‐blocking activity of the SIT‐induced polyclonal IgG response to assess the overall contribution to clinical efficacy.

Our findings raise the question whether there may be differences regarding cross‐reactivities of IgE and SIT‐induced IgG antibodies. At present, this question cannot be answered. However, one may speculate that in the case of SIT‐treated patients, differences in epitope specificities may be due to IgE responses induced by folded allergens and mainly conformational epitopes, whereas injection of adjuvant‐bound allergens also induces IgG responses against sequential (*i.e*. linear) epitopes. As mAb102.1F10 unlike allergic patients' IgE showed reactivity with the unfolded C‐terminal Phl p 7 peptide, it is quite likely that this antibody resulted from the induction of a *de novo* immune response against the unfolded adjuvant‐bound allergen in the course of SIT and that this explains its different binding behaviour.

In conclusion, we think that our molecular analysis of the SIT‐induced IgG_4_ antibody provides an example that SIT with cross‐reactive allergen does not always induce cross‐reactive and cross‐protective IgG antibodies.

## Author contribution

EG, SF and RV designed the project, analysed and interpreted the data and wrote the manuscript. EG, LKJ, MHS, KB and KF performed the experiments. WK, PV, SRD and HJG interpreted the data. PZ contributed with patients' sera. TG, MF‐T, MV and RB contributed with proteins. All authors provided critical review of the manuscript.

## Funding

Authors from the Medical University of Vienna were supported by Grants P23318‐B11, F4605, F4607 and F4611 of the Austrian Science Fund (FWF). KCL authors acknowledge financial support from the Department of Health via the National Institute for Health Research (NIHR) comprehensive Biomedical Research Centre award to Guy's & St Thomas' NHS Foundation Trust in partnership with King's College London and King's College Hospital NHS Foundation Trust.

## Conflict of interest

RV has received research grants from Biomay AG, Vienna, Austria, and Thermofisher, Uppsala, Sweden, and serves as a consultant for Biomay AG, Thermofisher and Fresenius Medical Care, Bad Homburg, Germany.

## Supporting information


**Figure S1** Inhibition of basophil activation induced by Phl p 7 and related EF‐hand allergens with mAb102.1F10.

**Table S1** Amino acid sequence identities (%) of Phl p 7 and related EF‐hand allergens.

**Table S2** Affinities of mAb102.1F10 to EF‐hand allergens.

**Table S3** Reactivity of mAb102.1F10 to Phl p 7 and related EF‐hand allergens in the presence or absence of calcium.

**Data S1** Description of Methods for surface plasmon resonance (SPR) measurements, for a RAST‐based assay to study mAb102.1F10 reactivity to EF‐hand allergens in the presence or absence of calcium and for basophil activation tests.
