# Supplementary material for: Epitope specificity determines cross‐protection of a SIT‐induced IgG4 antibody
Source: Allergy. 2015 Sep 30;71(1):36–46. doi: 10.1111/all.12710 (PMC4716291; doi:10.1111/all.12710)
Supplement: Supplementary file 4 — Table S1 Amino acid sequence identities (%) of Phl p 7 and related EF‐hand allergens. [file ALL-71-36-s004.docx]

Table S1

|  | ***Phl p 7*** | ***Aln g 4*** | ***Bet v 4*** | ***Bra r 1*** | ***Che a 3*** | ***Ole e 3*** | ***Ole e 8*** |
| --- | --- | --- | --- | --- | --- | --- | --- |
| *Phl p 7* | 100 |  |  |  |  |  |  |
| *Aln g 4* | 69 | 100 |  |  |  |  |  |
| *Bet v 4* | 68 | 91 | 100 |  |  |  |  |
| *Bra r 1* | 67 | 75 | 75 | 100 |  |  |  |
| *Che a 3* | 73 | 84 | 82 | 71 | 100 |  |  |
| *Ole e 3* | 68 | 82 | 80 | 71 | 81 | 100 |  |
| *Ole e 8* | 45 | 42 | 44 | 38 | 41 | 48 | 100 |
