# Supplementary material for: Epitope specificity determines cross‐protection of a SIT‐induced IgG4 antibody
Source: Allergy. 2015 Sep 30;71(1):36–46. doi: 10.1111/all.12710 (PMC4716291; doi:10.1111/all.12710)
Supplement: Supplementary file 5 — Table S2 Affinities of mAb102.1F10 to EF‐hand allergens. [file ALL-71-36-s005.docx]

Table S2

| ***Allergen*** | ***K_D_ [M]*** | |
| --- | --- | --- |
| *Phl p 7* | 2.11 x 10^-9^ |  |
| *Aln g 4* | 7.93 x 10^-6^ |  |
| *Bet v 4* | 6.26 x 10^-6^ |  |
| *Bra r 1* | 6.57 x 10^-6^ |  |
| *Ole e 3* | 6.18 x 10^-9^ |  |
| *Ole e 8* | ~7.06 x 10^-5^ |  |
