# Supplementary material for: Epitope specificity determines cross‐protection of a SIT‐induced IgG4 antibody
Source: Allergy. 2015 Sep 30;71(1):36–46. doi: 10.1111/all.12710 (PMC4716291; doi:10.1111/all.12710)
Supplement: Supplementary file 6 — Table S3 Reactivity of mAb102.1F10 to Phl p 7 and related EF‐hand allergens in the presence or absence of calcium. [file ALL-71-36-s006.doc]

Table S3

| ***Allergen*** | ***mAb102.1F10***  ***+ CaCl_2_***  ***[mean cpm]*** | ***mAb102.1F10***  ***– CaCl_2_***  ***[mean cpm]*** | ***Reduction [%]*** |  | ***Control IgG_4_***  ***+ CaCl_2_***  ***[mean cpm]*** |
| --- | --- | --- | --- | --- | --- |
| *Phl p 7* | 1164.4 | 101.4 | 91.3 |  | 32.3 |
| *Aln g 4* | 147.8 | 20.8 | 85.9 |  | 44.0 |
| *Bet v 4* | 189.1 | 30.6 | 83.9 |  | 49.3 |
| *Bra r 1* | 151.8 | 28.2 | 80.7 |  | 46.0 |
| *Che a 3* | 119.8 | 29.2 | 75.7 |  | 31.7 |
| *Ole e 3* | 2400.8 | 1602.8 | 33.2 |  | 25.8 |
| *Ole e 8* | 122.9 | 37.4 | 66.6 |  | 61.1 |
| *BSA* | 9.4 | 16.1 |  |  | 33.6 |
|  |  |  |  |  |  |
