# Supplementary material for: Epitope specificity determines cross‐protection of a SIT‐induced IgG4 antibody
Source: Allergy. 2015 Sep 30;71(1):36–46. doi: 10.1111/all.12710 (PMC4716291; doi:10.1111/all.12710)
Supplement: Supplementary file 7 — Data S1 Description of Methods for surface plasmon resonance (SPR) measurements, for a RAST‐based assay to study mAb102.1F10 reactivity to EF‐hand allergens in the presence or absence of calcium and for basophil activation tests. [file ALL-71-36-s007.docx]

**SUPPLEMENTARY INFORMATION**

**Epitope specificity determines cross-protection of a SIT-induced IgG_4_ antibody**

Short title: Characterization of a SIT-induced monoclonal IgG_4_

E. Gadermaier^1^, L. K. James^2^, M. H. Shamji^3^, K. Blatt^4^, K. Fauland^5^, P. Zieglmayer^6^, T. Garmatiuk^1^, M. Focke-Tejkl^1^, M. Villalba^7^, R. Beavil^2^, W. Keller^5^, P. Valent^4^, S. R. Durham^3^, H. J. Gould^2^, S. Flicker^1^, R. Valenta^1^

^1^Division of Immunopathology, Department of Pathophysiology and Allergy Research, Centre for Pathophysiology, Infectiology and Immunology, Medical University of Vienna, Vienna General Hospital, Vienna, Austria.

^2^Randall Division of Cell and Molecular Biophysics, King’s College London, SE11UL, United Kingdom.

^3^Allergy and Clinical Immunology, National Heart and Lung Institute, Imperial College London, London, SW3 6LY, United Kingdom.

^4^Division of Hematology and Hemostaseology, Department of Internal Medicine I, Medical University of Vienna, Vienna General Hospital, Vienna, Austria.

^5^Institute of Molecular Biosciences, University of Graz, Graz, Austria.

^6^Vienna Challenge Chamber, Allergy Centre Vienna West, Vienna, Austria.

^7^Departamento de Bioquımica y Biologıa Molecular I, Universidad Complutense de Madrid, Madrid, Spain.

Correspondence to:

Rudolf Valenta, MD

Division of Immunopathology

Department of Pathophysiology and Allergy Research

Centre for Pathophysiology, Infectiology and Immunology

Medical University of Vienna, Vienna General Hospital

Währinger Gürtel 18-20, A-1090 Vienna, Austria

Tel.: +43-1-40400-51080

Fax: +43-1-40400-51300

E-mail: rudolf.valenta@meduniwien.ac.at

**Materials and Methods**

**Surface plasmon resonance measurements**

In order to measure interaction kinetics and affinity between mAb102.1F10 and the EF-hand allergens Phl p 7, Aln g 4, Bet v 4, Bra r 1, Ole e3 and Ole e 8 by real-time detection surface plasmon resonance measurements (SPR) were performed using a Biacore T200 instrument. Purified recombinant IgG_4_ was first immobilised on a CM5 sensor chip (GE Healthcare Life Sciences) by amine coupling. Binding of recombinant allergens was measured using a 3-minute association phase and a 10-minute dissociation phase in a concentration series ranging from 25 μM to 10 nM. Experiments were performed at 25°C. Standard double referencing data subtraction methods were employed. Kinetic rate constants (Phl p 7 and Ole e 3) and equilibrium constants (Aln g 4, Bet v 4, Bra r 1 and Ole e 8) were determined using Biacore T200 Evaluation (GE Healthcare).

**Reactivity to EF-hand allergens in the presence and absence of calcium**

In order to investigate the reactivity and eventual calcium-dependence of mAb102.1F10 binding to recombinant Phl p 7, Aln g 4, Bet v 4, Bra r 1, Che a 3, Ole e 3, Ole e 8 and BSA as a control, nitrocellulose-dotted proteins (Schleicher & Schuell, Dassel, Germany) (1 µg/protein) were exposed to mAb102.1F10 or control IgG_4_ (1µg/ml) (Sigma-Aldrich, St. Louis, MO, USA) in the presence of either 0.1 mM CaCl_2_ or 5 mM EGTA. A rabbit antiserum specific for Phl p 7 was used to detect the dotted allergens (data not shown). Rabbit antisera raised against a non-relevant protein were used as controls to determine background reactions (data not shown). Bound human IgG_4_ antibodies were detected with a mouse monoclonal anti-human IgG_4_ antibody (PharMingen, San Diego, CA, USA) followed by ^125^I-labeled rabbit anti-mouse antibodies whereas rabbit antibodies were detected with ^125^I-labeled goat anti-rabbit antibodies (Perkin Elmer, Waltham, MA, USA). Binding of antibodies was quantified by gamma counting (Wizzard, Automatic Gamma Counter; Wallac, Uppsala, Sweden) and mean counts per minute (cpm)/dot are shown. Percent reduction of mAb102.1F10 binding due to calcium depletion was calculated according to the following formula: 100 – (cpm bound mAb102.1F10 in the presence of 5 mM EGTA * 100/cpm bound mAb102.1F10 in the presence of 0.1 mM CaCl_2_) (Table S3).

**Inhibition of basophil activation with Phl p 7 and related EF-hand allergens with mAb102.1F10**

To test whether mAb102.1F10 can inhibit patient’s basophil activation heparinized blood was collected from Phl p 7-allergic patients after informed consent was given. Blood samples (100 µl) were incubated for 15 minutes at 37°C with increasing concentrations of allergens (Phl p 7, Aln g 4, Bet v 4, Bra r 1, Che a 3, Ole e 3, Ole e 8; 1,28 pM – 4 nM) that had been pre-incubated overnight at 4°C with 66 nM of either mAb102.1F10 or control IgG_4_. For control purposes, cells were exposed to 1) IgG_4_ antibodies without allergen addition, 2) to 1 µg/ml of a monoclonal anti-IgE antibody (Immunotech, Marseille, France) or to buffer alone. Up-regulation of CD203c expression was determined by flow cytometry (1). Allergen-induced CD203c up-regulation was calculated from mean fluorescence intensities (MFIs) obtained with stimulated (MFI_stim_) and unstimulated (MFI_control_) cells and was expressed as stimulation index (SI) which is defined as MFI_stim_/MFI_control_. Mean SIs of triplicate determinations were calculated and background reactions of the IgG_4_ antibodies without allergen addition were subtracted (Fig. S1).

**Figures and Tables**

**Table S1** Amino acid sequence identities (%) of Phl p 7 and related EF-hand allergens aligned with each other are displayed.

**Table S2** Affinities of mAb102.1F10 to EF-hand allergens. Surface plasmon resonance measurements of mAb102.1F10 binding to Phl p 7 and to five homologous allergens (Aln g 4, Bet v 4, Bra r 1, Ole e 3, Ole e 8). Dissociation constants (K_D_s [M]) calculated by fitting the analysed data with a 1:1 binding model are shown.

**Table S3** Reactivity of mAb102.1F10 to Phl p 7 and related EF-hand allergens in the presence or absence of calcium. Mean cpm values and the percent reduction of mAb102.1F10 binding after depletion of calcium are shown. Background bindings by a control IgG_4_ binding are shown in the right column.

**Figure S1** Inhibition of basophil activation induced by Phl p 7 and related EF-hand allergens with mAb102.1F10. Blood samples from three Phl p 7-allergic patients (E1A: #2; E1B: #3; E1C: #19) were exposed to increasing doses of Phl p 7, Ole e 3, Aln g 4, Bet v 4, Bra r 1, and Che a 3 (x-axes: 1.28 pM – 4 nM) which had been pre-incubated with mAb102.1F10 (black bars) or control IgG_4_ (grey bars). Up-regulation of CD203c expression levels on basophils were determined by FACS analyses and are displayed as mean stimulation indices (SIs) after subtraction of SIs obtained by addition of antibodies without allergens (y-axes: SI +/- SD).

**References**

1. Hauswirth AW, Natter S, Ghannadan M, Majlesi Y, Schernthaner GH, Sperr WR, et al. Recombinant allergens promote expression of CD203c on basophils in sensitized individuals. *J Allergy Clin Immunol* 2002;**110**(1):102-109.
